# Radiogenomics for Glioblastoma Survival Prediction: Integrating Radiomics, Clinical, and Genomic Features Using Artificial Intelligence

**DOI:** 10.1007/s10278-025-01692-3

**Published:** 2025-10-08

**Authors:** Sebastian Buzdugan, Moona Mazher, Domenec Puig

**Affiliations:** 1 Department of Computer Engineering and Mathematics, Universitat Rovira I Virgili, Tarragona, Spain; 2 Hawkes Institute, Department of Computer Science, University College London, London, UK

**Keywords:** Glioblastoma, Radiogenomics, Machine learning, MRI, MGMT promoter methylation, Survival analysis, Radiomics features

## Abstract

Glioblastoma (GBM) remains one of the most formidable brain malignancies, characterized by a heterogeneous genetic profile that significantly influences patient prognosis. Per the 2021 WHO central nervous system classification, GBM is defined as an isocitrate dehydrogenase (IDH) wild-type diffuse astrocytic tumor. We analyzed two multi-institutional cohorts, UPENN-GBM (644 patients) and UCSF-PDGM (420 patients); after excluding the 116 and 42 IDH-mutant records, 528 and 378 wild-type cases remained for modelling. MGMT promoter methylation, present in 43% of GBM cases, correlates with enhanced survival outcomes, demonstrating a median survival of 504 days versus 329 days in unmethylated cases. In this study, we present a novel integration of imaging phenotypes, clinical characteristics, and molecular markers through the application of advanced machine learning methodologies, including Random Forest, XGBoost, LightGBM, and an optimized dense neural network (Dense NN). This integrative approach aims to refine survival prediction in GBM patients. MRI data were meticulously processed using the MRIPreprocessor tool and the radiomics Python library, facilitating the extraction of high-dimensional radiomic features. Our findings reveal that the proposed custom Dense NN model outperformed traditional tree-based algorithms, with the Dense NN achieving a concordance index (CI) of 0.86 on the UPENN-GBM dataset and 0.83 on the UCSF-PDGM dataset. The optimized Dense NN architecture features three hidden layers with 256, 128, and 64 units respectively, employing ReLU activation, L1/L2 regularization to mitigate overfitting, batch normalization to stabilize training, and dropout for improved generalization. This specific configuration was determined through hyperparameter tuning using techniques like RandomizedSearchCV. This integrative, non-invasive methodology provides a more nuanced assessment of tumor biology, thereby advancing the development of personalized therapeutic strategies. Our results underscore the transformative potential of artificial intelligence in delineating disease trajectories and optimizing treatment paradigms. Moreover, this research establishes a robust framework for future investigations in glioblastoma survival prediction, illustrating the efficacy of combining clinical, genetic, and imaging data to enhance prognostic accuracy within precision medicine paradigms for GBM patients.

## Introduction

Glioblastoma (GBM) is the most prevalent and aggressive form of primary brain tumor in adults, comprising 48.3% of all malignant brain tumors [1]. Despite advancements in multimodal therapeutic strategies, including surgical resection, radiotherapy, and chemotherapeutic agents, the prognosis for GBM remains highly unfavorable. The median overall survival for GBM patients is a mere 12–15 months, with a dismal 5-year survival rate of only 10% [2]. This grim prognosis is attributed to the tumor’s rapid growth, infiltrative nature, and resistance to conventional therapies, which collectively contribute to its challenging management [3].

Reliable survival prediction in GBM is crucial for several reasons. It facilitates patient counseling, aids in the design and interpretation of clinical trials, and enables clinicians to make evidence-based decisions regarding treatment [4]. Additionally, accurate survival prediction models can guide risk stratification strategies, thereby potentially enhancing patient outcomes and optimizing healthcare resources [5].

Recent advances in genomics have underscored the importance of genetic and epigenetic alterations in GBM biology. Since 2021, the WHO classifies isocitrate dehydrogenase (IDH)-mutant high-grade gliomas separately as “astrocytoma, IDH-mutant, grade 4,” while GBM is reserved for IDH-wild-type tumors [6, 7]. In the present work we analyzed only the IDH-wild-type subset of each cohort (UPENN 528/644 and UCSF 378/420 cases); the small mutant fraction was excluded because it no longer falls under the GBM definition [8, 9].

Another critical genetic marker is the methylation of the O6-methylguanine-DNA methyltransferase (MGMT) promoter [10]. MGMT plays a pivotal role in repairing DNA damage caused by alkylating agents such as temozolomide. Methylation of the MGMT promoter results in reduced expression of the MGMT enzyme, thereby enhancing tumor cell susceptibility to chemotherapy [11, 12]. Patients with MGMT promoter methylation exhibit improved responses to temozolomide, with a median overall survival of 21.7 months compared to 12.7 months for those without methylation [13, 14]. The use of these genetic markers in personalized therapy offers the potential for more aggressive treatment in patients with favorable markers and inclusion in experimental trials for those without [15].

In recent years, radiogenomics has emerged as a promising approach for non-invasive tumor characterization, linking imaging phenotypes with genetic and molecular profiles. Radiogenomics involves the analysis of quantitative imaging features (radiomics) to infer underlying genetic and molecular traits of tumors [16, 17]. Leveraging advanced machine learning and deep learning algorithms, radiogenomics has demonstrated potential in predicting key genomic alterations, MGMT promoter methylation, and other critical markers in glioblastomas [18, 19]. For instance, studies have shown that multimodal MRI-based radiomics can predict core signaling pathways and genomic alterations in GBM, correlating with patient outcomes [20, 21].

Despite these advances, the full potential of radiogenomics for GBM survival prediction remains underexplored. Most research to date has focused on individual data modalities, either genomics or radiomics, without integrating these modalities to leverage complementary information [22, 23]. Furthermore, there has been limited investigation into the effectiveness of sophisticated ensemble machine learning techniques in this context.

In order to improve survival prediction for patients with glioblastoma (GBM), this study fills in current gaps in the field by creating an extensive radiogenomics framework. We use a variety of cutting-edge machine learning methods, such as XGBoost, Random Forest, LightGBM, and a specially designed dense neural network, to combine clinical and genetic data with radiomics properties from magnetic resonance imaging (MRI). To demonstrate the potential of radiogenomics to significantly advance precision medicine for GBM patients, we aim to show that this integrated, multimodal approach offers superior predictive accuracy when compared to single-modality analyses or traditional linear regression models.

## Material and Methods

The methods are presented in the chronological order of the study: dataset curation, image normalization, radiomic extraction, feature reduction, integration of clinical and molecular data, model training with cross-validation, and performance assessment.

### Datasets

Two publicly accessible cohorts, UCSF-PDGM and UPENN-GBM, were analyzed independently in this study. Each cohort supplies multi-parametric MRI (mpMRI; T1-weighted, contrast-enhanced T1, T2-weighted, and FLAIR), clinical variables, and, for UCSF-PDGM, molecular annotations. To comply with the 2021 World Health Organization classification, only IDH-wild-type glioblastoma cases were retained, yielding 378 of 420 cases in UCSF-PDGM and 528 of 644 cases in UPENN-GBM. Within each cohort, the data were randomly divided into training, validation, and test subsets in a 75:20:5 proportion (UCSF-PDGM, 284/75/19; UPENN-GBM, 396/106/26). Test sets were held out during model development and hyper-parameter tuning.

**Fig. 1:**
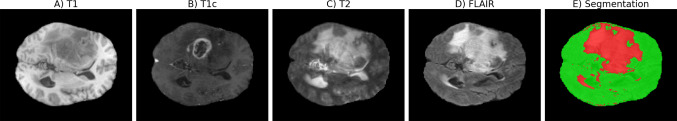
Axial mpMRI slices from a representative glioblastoma case. **A** T1-weighted image showing anatomical structure. **B** Contrast-enhanced T1 image displaying ring enhancement within the tumor core. **C** T2-weighted image showing peri-tumoral edema. **D** FLAIR image showing lesion conspicuity after cerebrospinal-fluid suppression. **E** Segmentation overlay: whole tumor (green) and enhancing core (red), the region used for radiomics feature extraction

The complementary information captured by the four mpMRI sequences and the expert tumor segmentation is illustrated in Fig. 1.

#### UPENN-GBM Dataset

The UPENN-GBM cohort comprises multi-parametric MRI volumes from 644 glioblastoma patients treated at the University of Pennsylvania Health System. Each case provides four co-registered sequences in NIfTI format (T1-weighted, contrast-enhanced T1, T2-weighted, and FLAIR), accompanying clinical variables such as age, sex, and overall survival, and expert tumor masks that delineate enhancing core, non-enhancing core, and peri-tumoral edema. Genomic information is limited to an IDH-status flag, which was used to exclude mutant tumors; MGMT-promoter methylation status is not reported. After filtering, 528 IDH-wild-type cases remained for downstream analysis. All MR volumes retain skull structures but are spatially normalized to a common reference space as supplied by the dataset curators [24]. Only voxels within the enhancing-core subregion were used for radiomic feature extraction, consistent with the pipeline described in the “Radiomics Feature Extraction” section.

#### UCSF-PDGM Dataset

The UCSF-PDGM cohort contains pre-operative mpMRI studies from 420 patients with diffuse glioma who underwent treatment at the University of California, San Francisco. Imaging data include the same four sequences as UPENN-GBM, provided skull-stripped and rigidly aligned by the dataset authors. Comprehensive clinical metadata accompany each case, together with molecular annotations for IDH1 mutation status and MGMT-promoter methylation status. Restricting the cohort to IDH-wild-type glioblastoma as defined in the 2021 WHO classification yielded 378 usable cases. As with UPENN-GBM, radiomic analysis was confined to the enhancing-core voxels delineated in the expert segmentation masks.

#### Comparison of UCSF-PDGM and UPENN-GBM Datasets

Demographic and clinical baselines and the statistical tests applied are summarized below. The sole systematic difference is molecular annotation: UCSF-PDGM records MGMT-promoter methylation in addition to IDH1 status, whereas UPENN-GBM supplies IDH1 status only.

Table 1 presents sex ratios, age distributions, and overall-survival (OS) medians. Three standard statistical tests were applied.

**Table 1:** Patient demographic data

Characteristic	UCSF dataset	UPENN dataset
No. of patients	420 (39%)*	644 (61%)*
Sex (*p* = 0.553)**		
Male	255 (61%)*	387 (60%)*
Female	165 (39%)*	257 (40%)*
Age (*p* = 0.909)**		
Age ranges	17–94	20.74–88.5
Median	61.0 (52.0–69.0)^†^	63.23 (55.93–71.43)^†^
Mean	59.540 (58.225–60.856)^†^	62.933 (62.017–63.849)^†^
OS (*p* = 0.978)**		
OS days ranges	6.0–4177.0	3–6109
Median	394.0 (186.5–676.0)^†^	376.0 (167.0–618.25)^†^
Mean	513.386 (469.132–557.639)^†^	496.290 (457.042–535.538)^†^

*Data in parentheses are percentages

**Data in parentheses are *p*-values indicating the level of statistical significance

^†^Data in parentheses are 95% confidence intervals, providing a range within which the true population parameter is expected to lie with 95% certainty

^‡^Analytic subset: Only the IDH-wild-type cases were used for modelling (UCSF = 378/UPENN = 528). All counts and summary statistics in this table reflect the full cohorts (UCSF = 420, UPENN = 644)

The chi-square test was used to assess the difference in sex distribution between the two datasets (Eq. 1). The *p*-value was 0.553.

1 $${X}^{2}= \sum \frac{{\left(O-E\right)}^{2}}{E}$$

The two-sample *t*-test compared mean ages between cohorts (Eq. 2). The *p*-value was 0.909.

2 $$t =\frac{ {\overline{x} }_{1}- {\overline{x} }_{2}}{\sqrt{{s}^{2}\left(\frac{1}{{n}_{1}}+\frac{1}{{n}_{2}}\right)}}$$

The log-rank test compared survival distributions (Eq. 3). The *p*-value was 0.978.

3 $${X}^{2}=\sum \frac{{\left(O-E\right)}^{2}}{E}$$

Radiomic shape traits such as elongation, flatness, and sphericity are available in both datasets, enabling comparable feature engineering.

#### Data Harmonization and Modelling Rationale

Each cohort (UCSF-PDGM and UPENN-GBM) was pre-processed and analyzed independently. Within each dataset, all MRI volumes were rigidly registered to a common anatomical template and intensity standardized using identical parameters. Training, validation, and test splits were defined only within the originating cohort, and every model was fitted exclusively to its own institutional data. Because no images were pooled across institutions, scanner-specific variability remained confined to the cohort of origin.

### Image Preprocessing

The MRIPreprocessor tool (https://github.com/ReubenDo/MRIPreprocessor) was utilized to preprocess MRI images from both datasets, following a multi-step pipeline. Initially, N4 bias field correction was applied to correct intensity non-uniformity in the MRI images. Next, HD-BET was employed to eliminate non-brain tissues, focusing specifically on the brain tissue. Intensity normalization was achieved through histogram matching to ensure uniform intensity values across different scans. The scans were then co-registered to a common reference space (MNI space, 1 × 1 × 1 mm) using ANTsPy. Finally, zero padding was removed from the skull-stripped images to concentrate on the brain region [25].

**Fig. 2:**
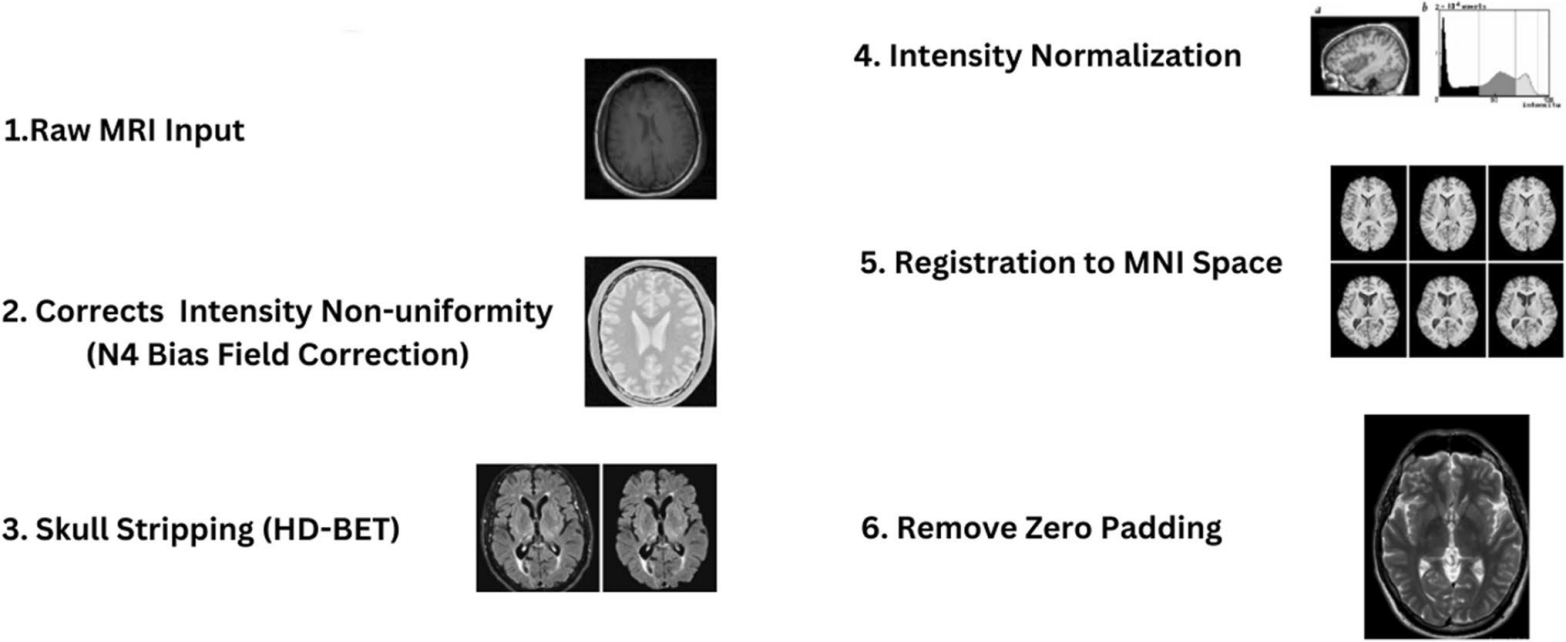
Visualization of MRI preprocessing workflow

Figure 2 represents a systematic procedure for preparing MRI data, demonstrating the progressive conversion of MRI pictures via several phases. The workflow comprises the subsequent stages: (1) The raw MRI scan is displayed; (2) intensity non-uniformity is corrected using N4 bias field correction to ensure uniformity in intensity levels throughout the image; (3) skull stripping is performed to isolate the brain tissue from the MRI; (4) intensity normalization is applied to standardize intensity values for consistency across different scans; (5) registration to MNI space is performed to align the brain images to a standard template for comparison; and (6) zero padding is removed to eliminate any unnecessary padding around the image. Every step is accompanied by a graphical depiction of the MRI data following the relevant processing stage, therefore offering a lucid perspective of the modifications implemented during preprocessing.

### Radiomics Feature Extraction

Radiomics features were extracted directly from T1, T1c, T2, and FLAIR MRI modalities using the PyRadiomics library (v3.1.0), in compliance with the Imaging Biomarker Standardization Initiative (IBSI) guidelines. A total of 116 features were extracted, comprising 14 shape descriptors, 18 first-order intensity statistics (e.g., mean, standard deviation, skewness, kurtosis), and 84 texture features derived from gray-level co-occurrence (GLCM), gray-level run length (GLRLM), gray-level size zone (GLSZM), and neighborhood gray-tone difference (NGTDM) matrices. All features were extracted exclusively from voxels within the enhancing-core mask; edema and non-enhancing core regions were excluded [26] (Table 2).

**Table 2:** Selected radiomic features extracted from UCSF-PDGM and UPENN-GBM datasets

Feature name	Category	Description
original_shape_Elongation	Shape	Measures the elongation of the tumor shape along its major axis
original_shape_Flatness	Shape	Quantifies the flatness of the tumor shape
original_shape_Volume	Shape	Total volume of the tumor
firstorder_Mean	Intensity	Average intensity of the tumor region
firstorder_StandardDeviation	Intensity	Standard deviation of voxel intensities within the tumor
texture_glcm_Contrast	Texture	Measures the contrast within the texture using gray-level co-occurrence matrix
texture_glcm_Correlation	Texture	Assesses the correlation between neighboring voxel intensities
texture_glszm_SmallAreaHighGrayLevelEmphasis	Texture	Evaluates the distribution of small areas with high gray-level values within the tumor
texture_ngtdm_Busyness	Texture	Assesses the level of busyness or complexity in the texture of the tumor
firstorder_Skewness	Intensity	Measures the asymmetry of the intensity distribution within the tumor region
texture_glszm_ZoneEntropy	Texture	Quantifies the randomness or complexity of gray-level zones within the tumor
original_shape_Sphericity	Shape	Measures how spherical the tumor shape is
firstorder_Kurtosis	Intensity	Evaluates the peakedness of the intensity distribution within the tumor region
texture_glrlm_LongRunEmphasis	Texture	Measures the distribution of long runs of consecutive voxels with the same gray level
texture_ngtdm_Coarseness	Texture	Quantifies the coarseness of the tumor texture

A representative subset of the extracted radiomic features. The full list of 116 features is available upon request

All features underwent identical preprocessing across both datasets. Numerical features were normalized using *scikit-learn* v1.4 StandardScaler, features with near-zero variance were removed using VarianceThreshold, and categorical variables were one-hot encoded [27].

In addition, an unsupervised variable (num_Cluster) was generated by applying *k*-means clustering (*k* = 4, selected via the elbow method) to the training features. Each case was assigned a cluster label (0–3), which was included as a categorical covariate in subsequent modelling.

### Radiomics Feature Selection

Strict feature selection was used to enhance model performance and reduce redundancy in the radiomic data. First, a univariate filter was applied: SelectKBest with the f-regression score retained the 30 voxel-wise features that showed the strongest (*p* < 0.05, Benjamini–Hochberg corrected) linear association with overall survival days.

Second, a model-based filter was run inside each cross-validation loop. A 500-tree Random Forest was fitted to the current training fold, and SelectFromModel kept only those variables whose mean decrease in impurity lay above the empirical 75th percentile of the importance distribution. This step yielded ≤ 50 predictors per fold (28 for UCSF-PDGM; 26 for UPENN-GBM).

Downstream models included Random Forest, XGBoost, and LightGBM; additional algorithms were also tested (see “Results”). SHapley Additive exPlanations (SHAP) values were computed on the final trained models using held-out predictions to obtain feature attribution scores in a model-agnostic manner.

### Genomic and Clinical Data Integration

Genomic and clinical covariates were incorporated to complement the radiomic feature set. In the UCSF-PDGM dataset, MGMT-promoter methylation status was available for all cases and included as a feature. In contrast, the UPENN-GBM cohort did not report MGMT, and no genomic covariate was available. IDH1 mutation status was consulted solely for filtering, and because only IDH-wild-type tumors were retained, the IDH1 column was removed before modelling.

Radiomic features were then combined with MGMT status (when available) and the core clinical variables (age, sex, and extent of resection) to form the final training matrices. Each dataset was modelled independently. Within each dataset, missing values were imputed using scikit-learn v1.4 SimpleImputer. Numeric variables were filled with the median value, and categorical variables with a constant placeholder (“missing”). This procedure ensured a complete and internally consistent feature set for each cohort prior to model training.

### Proposed Architecture

**Fig. 3:**
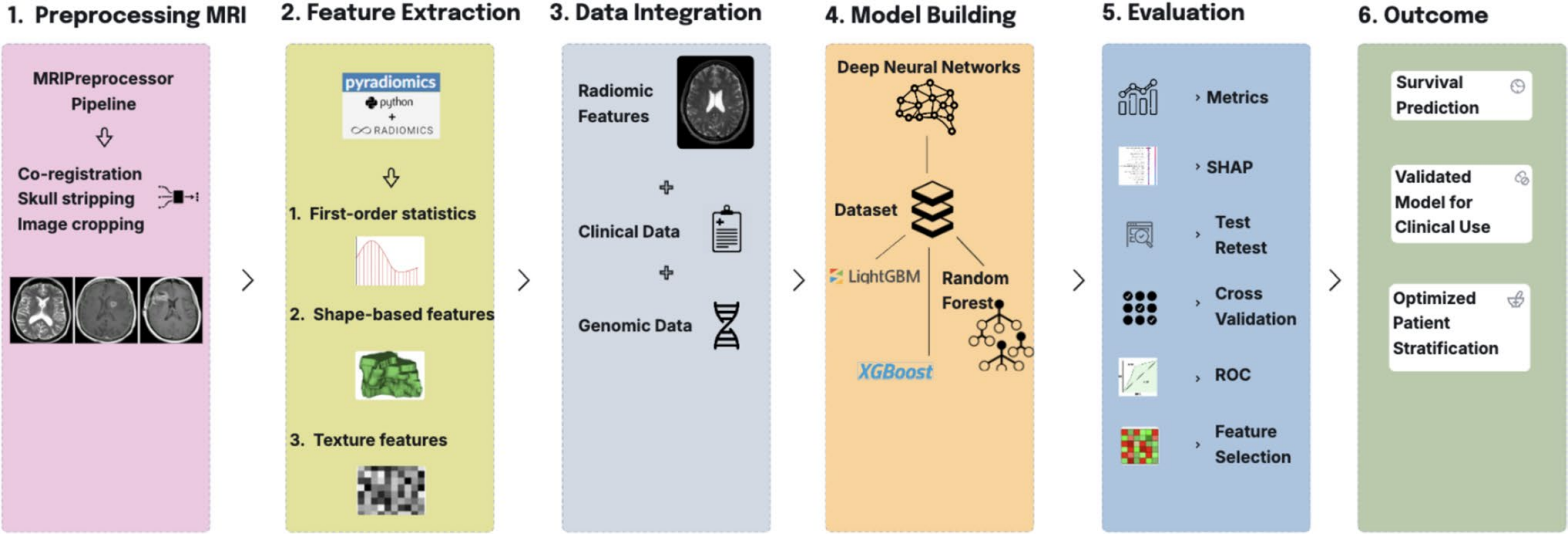
Workflow of radiomics and genomics analysis for survival prediction in neuro-oncology

Figure 3 illustrates the six main stages of the pipeline for forecasting survival results in neuro-oncology. (1) Data acquisition and preprocessing encompass the collection of multimodal MRI scans, clinical, and genomic data, followed by preprocessing procedures including co-registration, skull stripping, and image cropping. (2) Feature extraction entails the utilization of the PyRadiomics library to extract radiomic features, including shape, texture, and first-order statistics, as well as genomic and clinical qualities. (3) Data integration synthesizes the obtained radiomic characteristics with clinical and genetic data to form a cohesive dataset. (4) Model building for survival outcome prediction entails training Random Forest, XGBoost, LightGBM, and neural networks utilizing these integrated features. (5) Evaluation evaluates the model’s performance in terms of features, test–retest reliability, cross-validation, and ROC curves using metrics such as *R*^2^, MSE, MAE, and CI, in addition to SHAP analysis. (6) Outcome validates models to provide predictions of survival, facilitate optimal patient stratification, and assist in clinical selection.

Data preparation comprised (i) radiomic extraction with PyRadiomics (first-order, shape, GLCM/GLRLM/GLSZM/GLDM/NGTDM textures across T1, T1c, T2, and FLAIR); (ii) univariate filtering and tree-based importance ranking (SelectKBest, *f*-regression; Random Forest importance); and (iii) integration with clinical covariates (age, sex, GTR) and molecular markers (MGMT, IDH1). Missing values were imputed (median for numeric, “missing” for categorical), numerical features were z-scaled, and categorical variables one-hot encoded.

Each dataset was modelled separately using stratified train/validation/test splits (75%/20%/5%) with stratification based on survival time quintiles. For neural network architectures, fivefold cross-validation was employed within the training set to assess model stability. Hyperparameters for tree-based models (Random Forest, XGBoost, LightGBM) were optimized using RandomizedSearchCV with dataset-specific parameter grids.

To capture non-linear relationships, we implemented two deep-learning models trained on the same pre-processed feature matrix. The first, a dense neural network (Dense NN), contains three fully connected layers with 256, 128, and 64 units, each followed by a ReLU activation function, batch normalization, and a dropout layer with a rate of 0.40; kernel weights are regularized with combined L1 and L2 penalties (10^−4^ and 10^−3^, respectively). The second model adopts a wide-and-deep structure: a deep pathway of two hidden layers with 128 and 64 units is concatenated with a linear “wide” pathway before the final output node. To improve robustness, we also formed an ensemble by averaging the predictions of the Dense NN and the wide-and-deep network.

The concatenated radiomic, clinical, and genomic feature vector is fed into the Dense NN, which propagates through the three hidden layers described above and terminates in a single linear neuron that outputs predicted survival time. Optimization uses Adam with a learning rate of 6.25 × 10^−5^ for the Dense NN and 2.5 × 10^−4^ for the wide-and-deep network, with a mini-batch size of 32 and a maximum of 300 epochs. Early stopping halts training when validation loss fails to improve for 20 epochs, whereas a ReduceLROnPlateau scheduler lowers the learning rate by 90% after ten stagnant epochs. The set of weights that yields the best validation concordance index is preserved for final testing.

Hyperparameter optimization for tree-based models employed RandomizedSearchCV with dataset-specific parameter grids, evaluated using validation set performance. Neural network architectures underwent manual hyperparameter tuning with predefined learning rates optimized through systematic evaluation. All hyperparameter selection used validation sets; test sets were not used for tuning. Metrics were computed on held-out test sets.

Predictor sets were dataset-specific. UPENN-GBM models received 112 radiomic variables plus clinical covariates (age, sex, extent of resection > 90%). UCSF-PDGM models used the same input block and additionally incorporated MGMT-promoter methylation status.

Performance was quantified on held-out test sets using concordance index as the primary endpoint, supplemented by *R*^2^, mean squared error, and mean absolute error. For binary classification tasks, area under the receiver operating characteristic curve (AUC) was calculated for 1-year and 2-year survival thresholds where applicable.

### Statistical Analysis

Model evaluation relied on scikit-learn (v1.4) for cross-validation and metric computation and on lifelines (v0.28) for survival-specific analyses. Fivefold cross-validation was nested inside the training split to tune hyperparameters of Random Forest, XGBoost, LightGBM, and the custom dense neural network. Final evaluation was performed on the untouched 5% test sets; all reported metrics were derived from data not used for model training or tuning. Survival time and censoring flags were inherited directly from the source spreadsheets. In UCSF-PDGM, survival status was recorded as “1 = dead, 0 = alive,” while UPENN-GBM labelled “Alive” or “Lost-to-follow-up” as censored cases. The Kaplan–Meier curves and multivariable Cox models (Efron ties) were generated for each cohort to provide a descriptive survival baseline, although these statistical summaries were not incorporated into the machine-learning pipelines.

Model interpretability was assessed post hoc using SHapley Additive exPlanations (SHAP). SHAP values were computed on the held-out test predictions to enable consistent, model-agnostic analysis of feature contributions across the different algorithms evaluated.

## Results

The results are presented across four analyses: (i) predictive accuracy on independent test data, (ii) ablation experiments quantifying the contribution of each modality, (iii) comparisons with baseline machine-learning methods, and (iv) post hoc feature attribution analyses. Computational efficiency was also evaluated by recording relative training times.

### Performance Metrics

On held-out test sets, the dense neural network (Dense NN) achieved a concordance index of 0.83 in UCSF-PDGM and 0.86 in UPENN-GBM (Tables 3 and 4). Mean absolute error was 123 days in UCSF-PDGM and 130 days in UPENN-GBM, representing ~ 25% lower error compared with the best-performing tree-based ensemble. *R*^2^ values exceeded 0.75 in both cohorts, while ensemble methods plateaued near 0.65.

**Table 3:** Results of the UCSF-PDGM dataset (testing data)

Model	*R*^2^	MSE	MAE	C-index	AUC
Random Forest	0.57	104,500.40	236.82	0.74	0.81–0.88
XGBoost	0.64	93,500.91	220.16	0.75	0.83–0.88
LightGBM	0.67	104,000.53	230.76	0.73	0.80–0.88
Dense NN	0.77	55,568.35	145.58	0.83	0.92–0.93
Wide & Deep NN	0.67	81,015.45	200.16	0.76	0.86–0.91
Ensemble	0.74	67,093.38	170.78	0.81	0.91–0.93

**Table 4:** Results of the UPENN-GBM dataset (testing data)

Model	*R*^2^	MSE	MAE	C-index	AUC
Random Forest	0.79	49,734.96	165.09	0.79	0.88–0.98
XGBoost	0.57	94,011.57	225.57	0.78	0.87–0.97
LightGBM	0.76	55,192.17	175.76	0.78	0.88–0.97
Dense NN	0.91	24,946.76	122.88	0.86	0.93–0.99
Wide & Deep NN	0.87	34,377.12	143.27	0.80	0.86–0.99
Ensemble	0.90	27,260.96	129.09	0.84	0.91–0.99

### Ablation Studies

Ablation experiments were conducted to quantify the contribution of genomic, clinical, and radiomic features. Starting with the full feature set, Random Forest, XGBoost, and LightGBM were retrained after sequentially excluding one modality at a time, and performance was evaluated on held-out test sets.

Excluding genomic variables (IDH-wild-type indicator in both cohorts and MGMT-promoter methylation in UCSF-PDGM) reduced the concordance index from 0.84 to 0.75 for Random Forest, from 0.85 to 0.76 for XGBoost, and from 0.86 to 0.76 for LightGBM, corresponding to an average decrease of ~ 0.09. Excluding clinical covariates (age, sex) yielded the lowest performance, with C-index values of 0.56, 0.58, and 0.59. Excluding radiomic features reduced performance to approximately 0.64 across models.

### Comparison with Baseline Methods

UPENN-GBM and UCSF-PDGM were the two datasets used to compare the performance of the machine learning models to baseline techniques. Both datasets were divided into 75% training, 20% validation, and 5% held-out testing data. UCSF-PDGM models achieved higher concordance indices than the UPENN-GBM counterparts.

The models demonstrated consistent performance across training and testing sets. MSE and MAE values were low, and *R*^2^ and C-index scores were high.

Dense NN achieved the highest concordance index and lowest mean squared error compared to other models. The ensemble and Wide & Deep NN also performed strongly. In UCSF-PDGM, ROC-AUC values for 1-year and 2-year survival prediction reached 0.93 and 0.99, respectively. Dense NN and the ensemble maintained high test-set *R*^2^ alongside low MSE and MAE.

In addition, model performance was contextualized by comparing concordance indices with those reported in prior studies applying machine learning to glioblastoma survival prediction.

As shown in Tables 3, 4, and 5, the proposed multimodal framework achieved C-indices that are comparable to, and in several cases higher than, previously reported values across different datasets and modelling strategies.

**Table 5:** Comparative analysis of C-index values for predictive models in GBM patients

Model	Study	C-Index	Dataset	Notes
XGBoost	Lao [30]	0.75	SEER	Applied to a large-scale dataset with various ML models
XGBoost	Baid [31]	0.75	Clinical data	Employed RF-RFE for feature selection before XGBoost application
XGBoost	Tang [32]	0.709–0.884	MRI data	Varied C-index depending on subtype classification using XGBoost
Random Forest	Pei [33]	0.64–0.75	NCDB-Brain PUF	Outperformed other models in survival predictions at multiple time points
Random Forest	Luckett [34]	0.712	MAS/ADNI	Calibrated to estimate survival probability post-admission
LightGBM	Cerono [29]	0.696	Cancer Imaging Archive	Used joint intensity matrices from multimodal MRI data
LightGBM	Babaei Rikan [28]	0.76	NCDB-Brain PUF	Employed LightGBM for personalized survival predictions
XGBoost	Multimodality Multimodal Machine Learning Framework	0.80	UCSF	Comprehensive integration of genomic, radiomic, and clinical data
XGBoost	Multimodality Multimodal Machine Learning Framework	0.79	UPENN	Effective multimodal integration for enhanced predictive accuracy
Random Forest	Multimodality Multimodal Machine Learning Framework	0.79	UCSF	Highlighted the importance of multimodal data integration
Random Forest	Multimodality Multimodal Machine Learning Framework	0.84	UPENN	Combined genomic, radiomic, and clinical data for robust predictions
LightGBM	Multimodality Multimodal Machine Learning Framework	0.81	UCSF	Demonstrated the effectiveness of multimodal data integration
LightGBM	Multimodality Multimodal Machine Learning Framework	0.81	UPENN	Generalized well across datasets with multimodal data

### Impact of Multimodality

Model performance was re-evaluated after systematically excluding clinical, genomic, or radiomic features to quantify the contribution of each modality.

For Random Forest, the concordance index increased from 0.64 (clinical-only) and 0.59 (genomic-only) to 0.81 when all modalities were included in the UCSF-PDGM cohort. Radiomics alone achieved a concordance index of 0.76, and the full feature set provided a further 7% improvement.

XGBoost achieved a concordance index of 0.83, compared with 0.64 when genomics were omitted and 0.52 with only clinical variables. Removing radiomics reduced the concordance index to 0.77, an 8% decline relative to the full model.

LightGBM demonstrated the largest dependence on multimodal inputs. The full model achieved a concordance index of 0.81, compared with 0.49 for genomics-only and 0.61 for clinical-only. Excluding radiomics reduced performance to 0.74, a 9% decline compared with the multimodal configuration.

### SHAP and Feature Importance Analysis

**Fig. 4:**
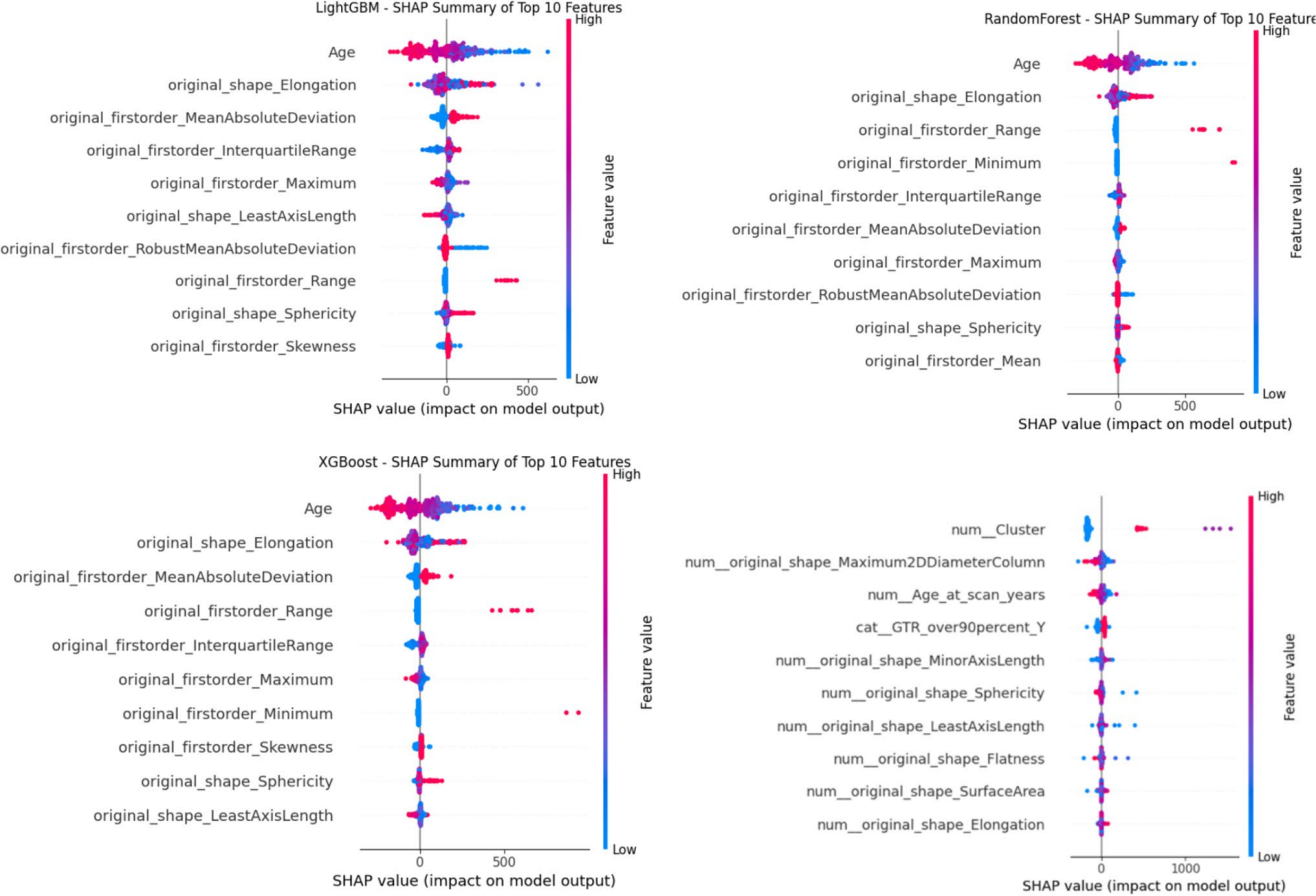
Combined SHAP summary plots for LightGBM, Random Forest, XGBoost, and dense neural network models

SHapley Additive exPlanations (SHAP) was applied to Random Forest, XGBoost, LightGBM, and dense neural network models to quantify the relative contribution of individual predictors. SHAP summary plots for the top ten features were generated for each model and combined into a single visualization (Fig. 4).

In the tree-based models (Random Forest, XGBoost, LightGBM), age consistently ranked as the most influential predictor. Shape-based radiomic descriptors, including elongation, sphericity, and maximum two-dimensional diameter, followed as the next most important variables.

In contrast, the dense neural network prioritized the clustering-derived variable (num_Cluster) as its dominant feature, with influence distributed across a broader set of radiomic and clinical covariates compared to the tree ensembles.

### Complexity Analysis

Training times were documented on a standard hardware platform to assess computational efficiency. Using the dense neural network (Dense NN) as the baseline (100% training time), tree-based models required substantially less computation, ranging from 5 to 15% of the Dense NN time. Among these, LightGBM was the fastest, followed by XGBoost, while Random Forest required the longest training time within the tree-based group.

The observed differences reflect inherent efficiency characteristics of the algorithms. LightGBM, for example, is designed with optimized histogram-based split finding, which reduces computational overhead relative to Random Forest and XGBoost. Although cross-validation increased total runtime across all models, the relative efficiency ranking remained consistent.

## Discussion

We next move from numerical evaluation to qualitative interpretation, analyzing how network design choices, data composition, and feature interactions converged to yield the observed gains in survival prediction.

### Interpretation of Results

Multimodal integration improved survival prediction relative to single-modality baselines, consistent with SHAP indicating complementary contributions from radiomics, MGMT, and clinical covariates. The slightly higher concordance observed in UCSF than UPENN is plausibly related to the availability of MGMT status in UCSF, although this requires confirmation. Gains appear driven by nonlinear cross-modal interactions, as SHAP shows broader feature influence in the neural model, while tree ensembles emphasize age and shape descriptors. This suggests that diverse modalities carry additive signal. Differences across cohorts and algorithms highlight sensitivity to data composition and underscore the need for external, prospective validation to assess generalizability. Overall, the findings support integrating imaging, molecular, and clinical data for risk stratification while acknowledging dataset heterogeneity and potential overfitting.

### Strengths and Limitations of the Proposed Framework

The principal strength of the proposed framework is the integration of radiomic, genomic, and clinical evidence, which captures latent interactions not accessible to single-modality or purely statistical approaches. As shown in Table 5, our framework achieved performance that is comparable to or higher than values reported in prior studies, underscoring the benefit of multimodal integration.

Despite these advantages, the framework has limitations. Its reliance on high-quality multimodal datasets may limit applicability in settings where such data are unavailable. Challenges related to overfitting and generalizability also remain, particularly in heterogeneous patient populations. Another limitation is that clustering-derived features (num_Cluster) depend on training data partitions, which may affect reproducibility. Finally, the framework has not yet been validated in prospective clinical trials, restricting immediate clinical translation.

### Clinical Implications

The proposed framework has significant clinical implications, particularly for personalized medicine. By providing more accurate survival predictions, it can aid clinicians in making more informed decisions regarding treatment planning and patient management. With its capacity to examine intricate patterns, the specially created deep learning model offers improved prediction estimates that may result in more precisely focused treatments. The integration of non-invasive radiogenomics could potentially reduce the need for biopsies, thus lowering patient risk and discomfort.

### Future Directions

While the models evaluated in this study demonstrated strong predictive capabilities through multimodal data integration, alternative approaches such as convolutional neural networks (CNNs), attention-based models, and principal component analysis (PCA) were not employed. CNNs are well-suited for image-based data but may not directly handle structured data comprising genomic, radiomic, and clinical features, limiting their applicability in this context. Attention-based models, although powerful for complex feature interactions, are prone to overfitting when applied to datasets with limited sample sizes, such as UCSF-PDGM (*n* = 378) and UPENN-GBM (*n* = 528). PCA, though effective in dimensionality reduction, may prioritize variance over clinically meaningful features, sacrificing interpretability, an attribute that is critical for building trust in clinical settings. These limitations underscore the rationale behind selecting ensemble learners and custom neural-network architectures, which balance predictive accuracy and interpretability.

These exploratory avenues complement, rather than replace, the present tree-ensemble + Dense-NN framework and will be tested under identical cross-validation protocols to ensure fair comparison.

Future research should focus on expanding the framework by incorporating additional data types, such as histopathological images or molecular markers, and developing more advanced models, including further advancements in custom deep learning architectures. Longitudinal studies tracking changes in imaging and genomic data over time could provide further insights into tumor progression and treatment response. Moreover, validating the framework in larger, more diverse patient populations is essential to ensure its broad applicability in clinical practice.

## Conclusion

This study demonstrates the effectiveness of integrating radiomic, clinical, and genomic features within multimodal machine learning frameworks to improve survival prediction in glioblastoma. The approach consistently achieved high predictive accuracy across independent cohorts, highlighting the added value of multimodality compared with single-modality models.

While these results support the role of AI-based prognostic tools in precision neuro-oncology, several limitations remain. The framework depends on high-quality datasets, carries risk of overfitting in smaller subsets, and requires external validation in diverse populations. Future work should focus on incorporating additional molecular markers, exploring advanced architectures, and conducting prospective clinical studies to strengthen reproducibility and enable translation into clinical practice.

## Data Availability

The datasets analyzed during the current study are publicly available.

## Code Availability

The analysis utilized publicly available tools including MRIPreprocessor (https://github.com/ReubenDo/MRIPreprocessor) and PyRadiomics (https://pyradiomics.readthedocs.io/).
